# 5-Fluorouracil Induces Diarrhea with Changes in the Expression of Inflammatory Cytokines and Aquaporins in Mouse Intestines

**DOI:** 10.1371/journal.pone.0054788

**Published:** 2013-01-30

**Authors:** Hiroyasu Sakai, Atsunobu Sagara, Kenjiro Matsumoto, Satoshi Hasegawa, Ken Sato, Maiko Nishizaki, Tetsuro Shoji, Syunji Horie, Takayuki Nakagawa, Shogo Tokuyama, Minoru Narita

**Affiliations:** 1 Department of Pharmacology, Hoshi University, Tokyo, Japan; 2 Laboratory of Pharmacology, Josai International University, Togane, Japan; 3 Department of Molecular Pharmacology, Graduate School of Pharmaceutical Sciences, Kyoto University, Kyoto, Japan; 4 Department of Clinical Pharmacy, School of Pharmaceutical Sciences, Kobe Gakuin University, Kobe, Japan; 5 Research promotion committee, Japanese Society for Pharmaceutical Palliative Care and Sciences, Tokyo, Japan; French National Centre for Scientific Research, France

## Abstract

Although the mechanisms of 5-fluorouracil (5-FU)-induced diarrhea remain unclear, accumulating evidence has indicated that changes in the mucosal immune system and aquaporins (AQPs) may play a role in its pathogenesis. Therefore, we investigated the possible changes in the gene expression of inflammatory cytokines and AQPs in the intestines of mice with 5-FU-induced diarrhea. In the present study, the expressions of mRNAs that encode inflammatory cytokines, TNF-α, IL-1β, IL-6, Il-17A and IL-22, were significantly increased throughout the entire colon of mice that exhibited diarrhea following 5-FU administration. In contrast, the gene expression of IFNγ was upregulated only in the distal colon. These increases were significantly reduced by the administration of etanercept. However, 5-FU-induced diarrhea was not recovered by etanercept. On the other hand, the genes for AQPs 4 and 8 were markedly present in the colon, and these expressions in the intestines were significantly decreased by treatment with 5-FU. These decreases were not reversed by etanercept. These findings suggest TNF-α neutralization had no effect on the acutely 5-FU-induced diarrhea and impaired AQPs but reduced dramatically several inflammatory cytokines.

## Introduction

The antimetabolite agent 5-fluorouracil (5-FU) is most commonly used as a chemotherapy drug in the treatment of various cancers, including colorectal and breast cancers [1]. Gastrointestinal (GI) mucositis is a common side effect of cancer chemotherapy for which there is no efficient treatment. It is currently the most significant dose-limiting toxicity of 5-FU treatment [2]. Previous studies have demonstrated that GI mucositis is a consequence of various processes, such as apoptosis, hypoproliferation, altered absorptive capacity and inflammatory response, and contributes to intestinal barrier dysfunction [2], [3]. In addition, cancer chemotherapy-induced intestinal mucositis increases the expression of proinflammatory-cytokines, such as TNF-α, IL-1β, and IL-6 [4], [5].

The recirculation of fluids in the GI tract is especially high during a meal, when water is secreted in the upper GI tract to allow the rapid osmotic balancing of intestinal contents, but is also continuously absorbed together with nutrients [6]. On average, the intestines absorb about 9.0 L/day [7]. Therefore, the absorption of water is one of the key functions of the intestines. The regulation of transepithelial fluid transport in the GI tract is based on ion transport and water transport by aquaporins (AQPs) [8]. AQPs constitute a family of small integral membrane proteins that are selectively permeable to water and driven by osmotic gradients [9], [10], [11], [12]. Thirteen AQP subtypes (AQPs 0, 1, 2, 3, 4, 5, 6, 7, 8, 9, 10, 11 and 12) have been cloned from mammals [13], [14], [15], [16]. AQPs 1, 3, 4, 5, 7, 8, 9 and 11 are localized in the intestines of humans [7], and AQPs 1, 3, 4, 7, 8, and 9 are localized in the intestines of mice [17], [18], [19], [20], [21]. It is widely thought that AQPs are involved in diseases that are characterized by alterations in water transport. It has been reported that a defect in the expression and/or function of AQPs underlies renal diabetes insipidus [22], brain edema [9], [23], dry eye [24] and food allergy-induced diarrhea [25].

Diarrhea is a common symptom of patients with inflammatory bowel disease (IBD), and a reduction in the expression of AQPs appears to be correlated with increased disease activity in patients with ulcerative and Crohn’s colitis [26]. The GI tract is capable of secreting large amounts of water, and the transepithelial hypersecretion of fluid is the basis of secretory diarrhea. However, defects in water absorption in the intestine are also important factors in the pathogenesis of diarrhea. The changes in AQP expression in diseases of the digestive system have been useful for understanding the functions of AQPs. However, little, if any, is known about the possible changes in the tissue levels of AQP expression in 5-FU-induced diarrhea.

To investigate the pathophysiological role of inflammatory cytokines and AQPs in 5-FU-induced diarrhea, we examined the possible changes in the gene expression of inflammatory cytokines and AQPs in the small and large intestines of mice under treatment with 5-FU. We also investigated the effect of the TNF-α inhibitor etanercept on the 5-FU-induced changes in the gene expression of inflammatory cytokines and AQPs in the intestines and on the development of diarrhea with 5-FU.

## Materials and Methods

### Animals

Male C57BL/6J mice (8–9 weeks of age, 23–27 g) were used. All experiments were approved by the Animal Care Committee at Hoshi University (Tokyo, Japan).

### Treatment Protocol

**Figure 1:**
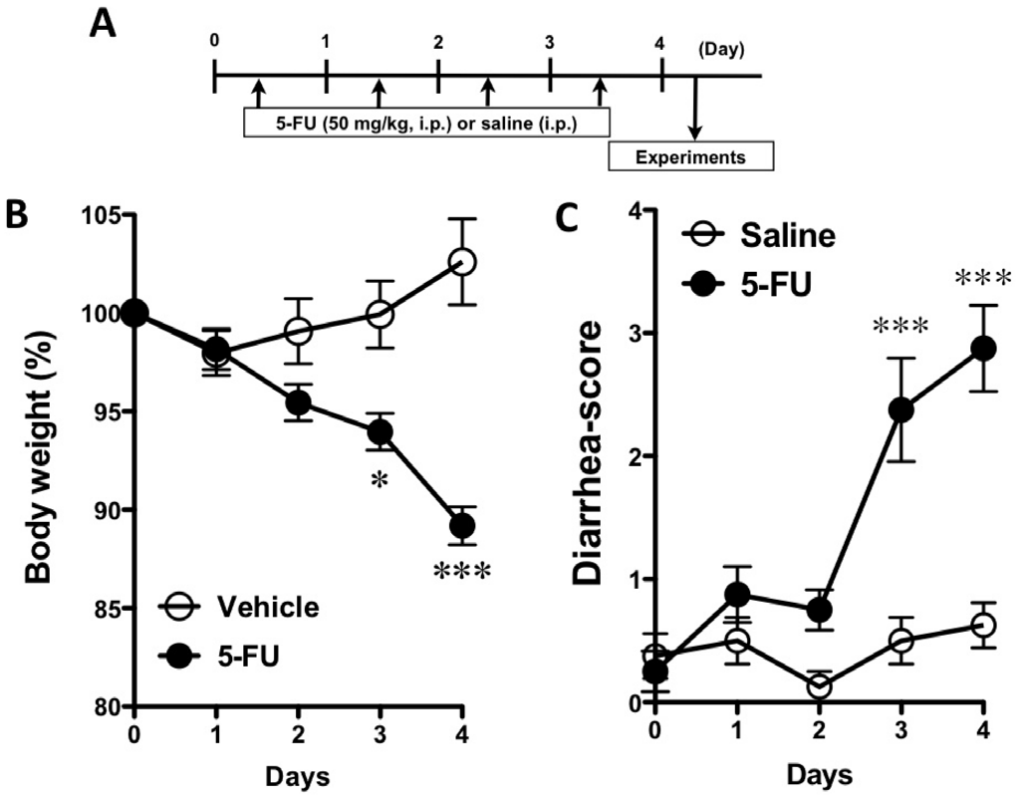
Schedule for the administration of 5-fluorouracil (5-FU) and the effect of 5-FU on body weight and the diarrhea-score. A) 5-FU (50 mg/kg, i.p.) or vehicle (saline, i.p.) was administered on Days 0–3. Twenty-four hours after the final administration of 5-FU, experiments were performed. B) Body weight decreased significantly under the repeated administration of 5-FU. C) Changes in the diarrhea-score during 5-FU-administration. The diarrhea-score significantly increased with 5-FU. Each point represents the mean ± S.E.M. of 6–9 mice. *p<0.05 and ***p<0.001 vs. vehicle (saline).

Mice were given a single intraperitoneal injection of of 5-fluorouracil (5-FU; 50 mg/kg) daily for 4 days, with saline (vehicle) used as a control (Figure 1A). Twenty-four hr after the final injection of 5-FU (Day 3), animals were killed under deep anesthesia with isoflurane, and the jejunum, ileum, proximal colon, transverse colon, and distal colon were removed, washed with cold saline, and stored in TRI Reagent™(Sigma-Aldrich) at −80°C. In mice treated with etanercept (Whyeth) etanercept (5 mg/kg) was administered subcutaneously 30 min before the administration of 5-FU on Days 0 and 3.

### Diarrhea Assessment

A diarrhea score was determined for each mouse. The severity of diarrhea was scored using the following scale, 0: normal (normal stool), 1: minimal (soft stool), 2: slight (slightly wet and soft stool), 3: moderate (wet and unformed stool with moderate perianal a staining of the coat), 4: severe (watery stool with severe perianal staining of the coat). The incidence of each diarrhea score (0 to 4) and the average diarrhea score were used to evaluate the severity of diarrhea.

### Real-time RT-PCR

mRNA levels of cytokines and AQPs were examined by real-time RT-PCR as described previously [27]. Briefly, total RNA was extracted from various tissues with a one-step guanidium–phenol–chloroform extraction procedure using TRI Reagent™(Sigma-Aldrich). cDNAs were prepared from total RNA (1.0 µg) by using QuantiTect Reverse Transcriptase (Qiagen, Germany) after incubation with gDNA wipeout buffer at 42°C for 3 min to remove contaminating genomic DNA. The reaction mixture (2 µL) was subjected to PCR (50 nM forward and reverse primers, Fast SYBR Green Mastermix; Applied Biosystems) in a final volume of 10 µL. The PCR primer sets used are shown in Table S1. The thermal cycle profile used was 1) denaturing for 30 s at 95°C, and 2) annealing for 30 s at 60°C. PCR amplification was performed for 40 cycles. Data are expressed as the expression relative to GAPDH mRNA as a housekeeping gene using the 2−deltadeltaCT method [27].

### Immunoblotting

Homogenates of distal-colonic tissue were prepared as follows. In brief, colonic tissues were removed and immediately soaked in ice-cold, oxygenated phosphate-buffered saline. They were carefully cleaned of adhering connective tissues, blood vessels and lung parenchyma under stereomicroscopy. The tissue was then homogenized in ice-cold T-PERTM Tissue Protein Extraction Reagent (Pierce). The tissue homogenate was centrifuged (1,000×g, 4°C for 15 min), and supernatants were stored at –80°C until use. To quantify AQP 4 and 8, Western blotting was performed. In brief, the samples (10 µg of total protein per lane) were subjected to 10–20% sodium dodecyl sulfate-polyacrylamide gel electrophoresis (SDS-PAGE). The proteins were then electrophoretically transferred to a PVDF (polyvinylidene difluoride) membrane. After being blocked with 3% bovine serum albumin, transferred PVDF membranes were incubated with the primary antibodies. Rabbit anti-AQP4 (1∶1000 dilution; Millipore) or mouse anti-AQP8 (1∶1000; Sigma-Aldrich) was used as a primary antibody. The membranes were then incubated with horseradish peroxidase-conjugated goat anti-rabbit immunoglobulin (Ig) G (1∶5000 dilution; GE Healthcare), and detection was performed with an ECL system. The housekeeping gene was detected on the same membrane by using monoclonal mouse anti-GAPDH (1∶5,000 dilution; Chemicon International) and horseradish peroxidase-conjugated sheep anti-mouse IgG (1∶5,000 dilution; GE Healthcare) to confirm that the same amounts of proteins were loaded. To normalize the AQP contents to GAPDH, the ratio of the corresponding AQP to GAPDH (AQP/GAPDH) was calculated as an index of AQP.

### Statistical Analysis

The statistical significance of differences was determined by an unpaired Student t-test or one-way analysis of variance (ANOVA) with the Bonferroni/Dunn post hoc-test. A value of p<0.05 was considered significant.

## Results

The body weight of mice was significantly decreased by the administration of 5-FU on Days 3 and 4 (Figure 1). The diarrhea-scores were significantly increased by the administration of 5-FU on Days 3 and 4 (p<0.001, Figure 1C).

**Figure 2:**
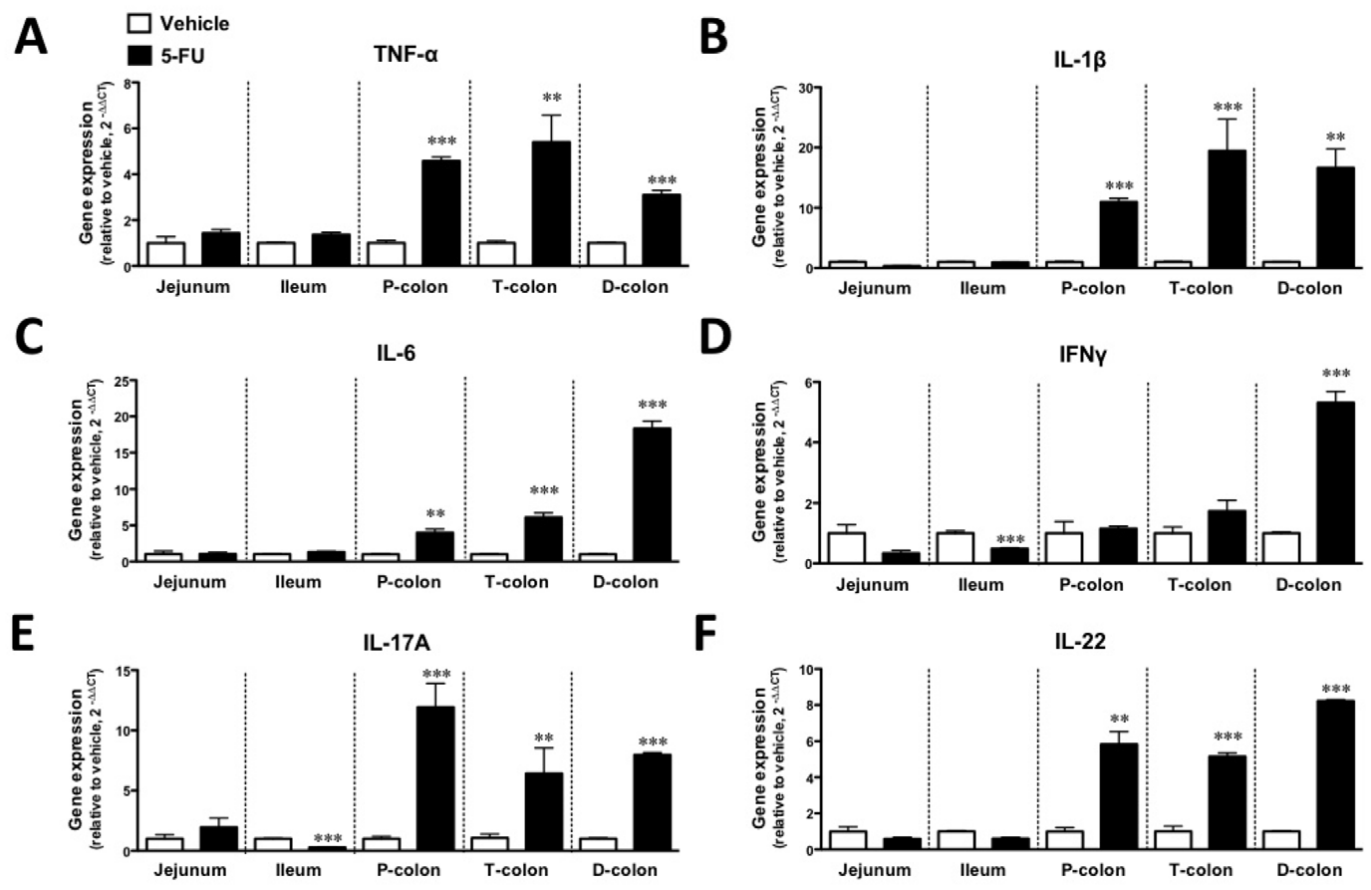
Changes in inflammatory cytokine gene expression in the intestines of mice treated with 5-FU. Treatment with 5-FU increased the gene expressions of TNF-α (A), IL-1β(B), IL-6 (C), IFNγ (D), IL-17A (E) and IL-22 (F) different parts of the colon. Each column represents the mean ±S.E.M. of 4–6 independent experiments. *p<0.05, **p<0.01 and ***p<0.001 vs. vehicle (saline).

To investigate the status of inflammation in the intestines, we examined the 5-FU-induced changes in the gene expression of inflammatory cytokines and cyclooxygenase 2 (COX2) in the jejunum, ileum, proximal colon, transverse colon and distal colon. As shown in Figure 2, the expressions of the TNF-α, IL-1β, IL-6, IL-17A and IL-22 genes were significantly upregulated by the intraperitoneal injection of 5-FU throughout the colon on Day 5 (p<0.01–0.001). Interestingly, the Th1-type cytokine IFNγ was significantly upregulated by 5-FU only in the distal colon (p<0.001), whereas INFγ and IL-17A were significantly downregulated in the ileum (p<0.001). However, no significant difference was observed in the expression of the COX2 gene among the regions of the colon (data not shown).

**Figure 3:**
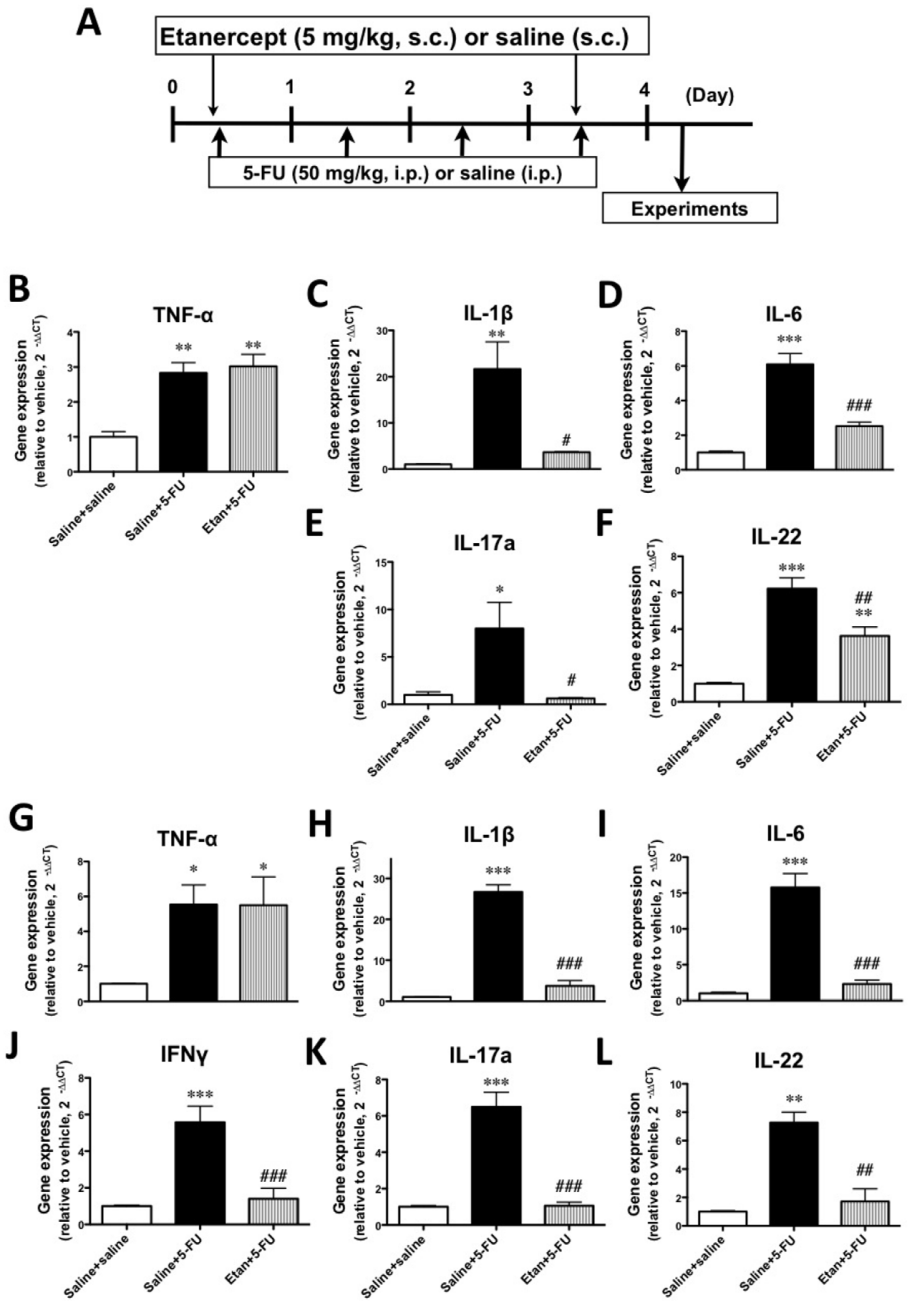
Schedule for the administration of the TNF-αinhibitor etanercept and the effects of etanercept on the 5-FU-induced upregulation of inflammatory cytokine gene expression in the colons of mice. A) Thirty minutes before the administration of 5-FU, etanercept (5 mg/kg, s.c.) or vehicle (saline, s.c.) was administered on Days 0 and 3. Twenty-four hours after the final administration of 5-FU, experiments were performed. The upregulated gene expressions of TNF-α (B and G), IL-1β (C and H), IL-6 (D and I), IFNγ (J), IL-17A (E and K) and IL-22 (F and L) by 5-FU were significantly inhibited by the administration of etanercept in the transverse (B-F) and distal (G-L) colons. Each column represents the mean ± S.E.M. of 4–6 independent experiments. *p<0.05, **p<0.01 and ***p<0.001 vs. Saline+saline. ^#^p<0.05, ^##^p<0.01 and ^###^p<0.001 vs. Saline +5-FU.

We next investigated the effect of the TNF-α inhibitor etanercept on 5-FU-induced changes in the gene expression of inflammatory cytokines. No significant changes in the gene expression of TNF-α, IL-1β, IL-6 and IL-17A were observed with the administration of etanercept alone. As expected, the 5-FU-induced increase in TNF-α gene expression in the colons of mice was not changed by treatment with etanercept. Under these conditions, the 5-FU-induced increases in the expressions of IL-1β, IL-6, IFNγ, IL-17A and IL-22 were significantly decreased in the transverse and distal colon with the administration of etanercept (p<0.05–0.001, Figure 3).

**Figure 4:**
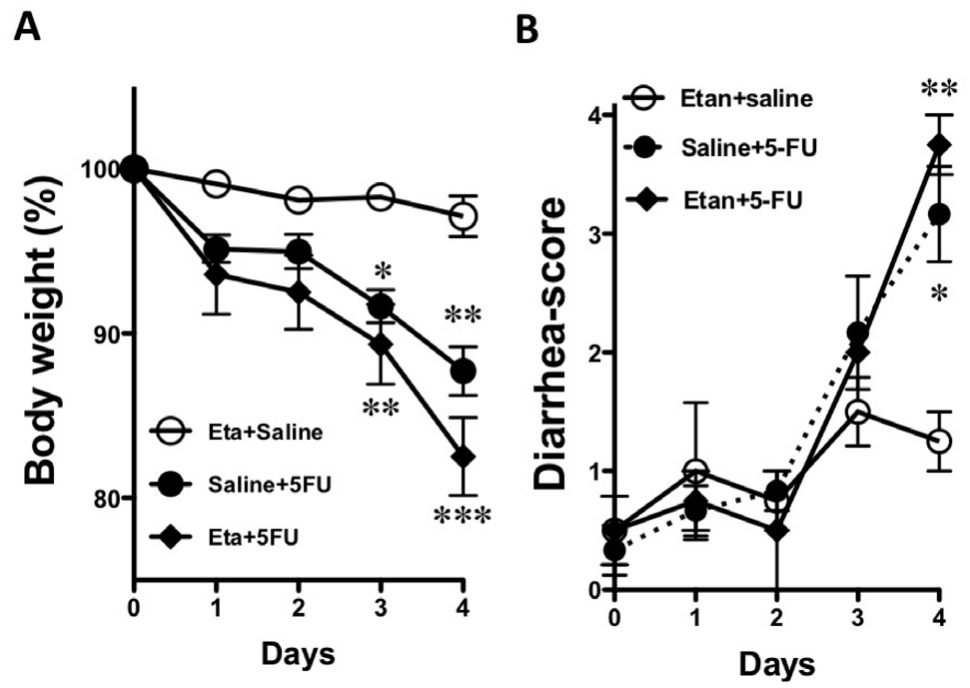
Effect of the administration of etanercept on the 5-FU-induced changes in body weight and the diarrhea-score. The 5-FU-induced changes in body weight (A) and the diarrhea-score (B) were not affected by etanercept. Each point represents the mean ± S.E.M. of 3–6 independent experiments. *p<0.05 and **p<0.01 vs. Etanercept+vehicle (Eta+saline).

As shown in Figure 4A, the 5-FU-induced loss of body weight was not recovered by the administration of etanercept. In addition, 5-FU-induced diarrhea was slightly, but not significantly, exacerbated by the administration of etanercept (Figure 4B).

**Figure 5:**
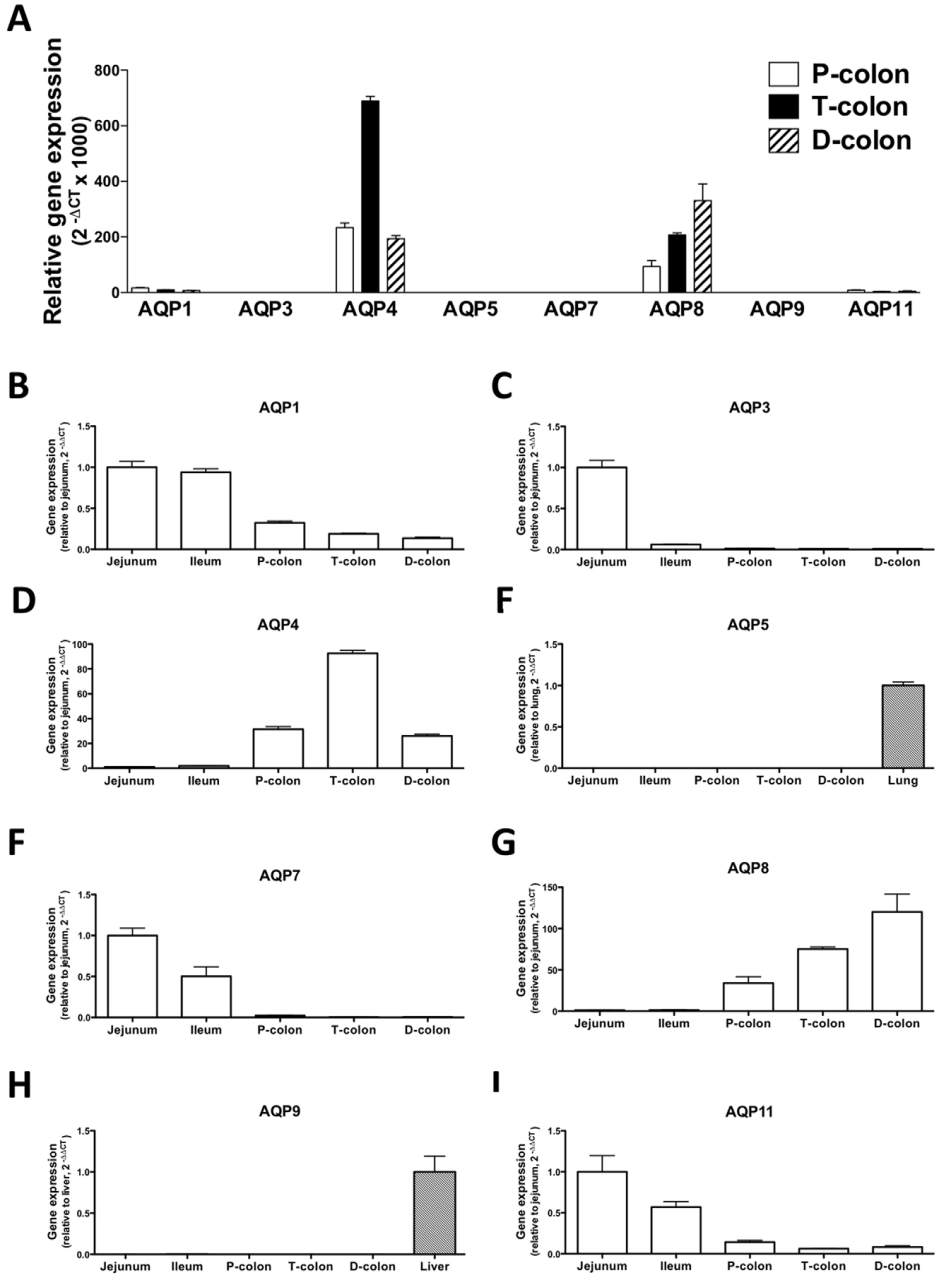
Distribution of aquaporin (AQP) gene expression in the intestines of the mice. The gene expression of AQPs 1, 3, 4, 5, 7, 8, 9 and 11 in the colon (A). The genes for AQP4 and 8 were clearly expressed in different parts of the colonGene expression is shown as 2-deltaCT values. The genes for AQPs 1 (B), 3 (C), 4 (D), 7 (F), 8 (G) and 11 (I) were expressed in the intestines. B-D and F-I). Gene expression is shown as the fold-amount relative to that in the jejunum. E and H) There is very little gene expression for AQPs 5 (D) and 9 (G) in the intestines of mice. Therefore, the expression of these genes is shown as the fold-amount relative to a positive control (mouse lung or liver). Each column represents the mean ± S.E.M. of 3 independent experiments.

The human intestine has been reported to contain AQPs 1, 3, 4, 5, 7, 8, 9 and 11 [7]. However, the expression of AQPs 5 and 9 has not been reported in murine intestine. In the present study, we investigated the gene expression of AQPs 1, 3, 4, 5, 7, 8, 9, and 11 in the intestines of normal-naïve mice. As shown in Figure 5, the gene expressions of AQP4 and 8 were clearly present in the colons. On the other hand, the 2-deltaCT values of AQP5 and AQP9 in the colon were markedly low (Figure 5A). The 2-deltadeltaCT values in the intestines were much lower than those in the lung and liver, which were used as a positive control [28], [29] (Figures 5E and H). The gene expression levels of AQPs 1, 7 and 11 decreased from the jejunum to the distal colon. In contrast, AQP8 expression increased from the distal colon to the jejunum. The gene expression of AQP3 predominantly existed in the jejunum, whereas AQP4 was especially prominent in the transverse colon.

**Figure 6:**
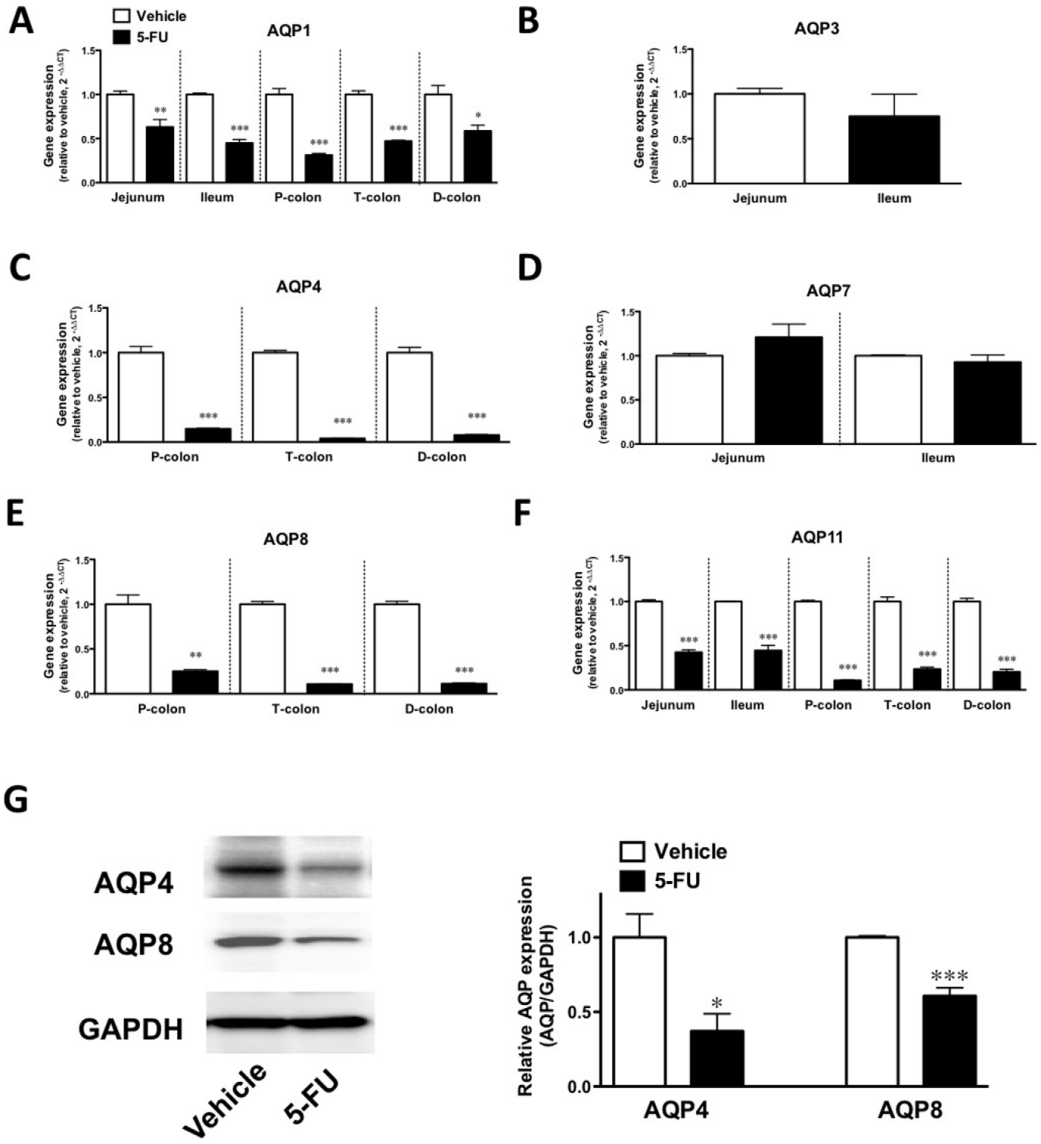
Effect of the administration of 5-FU on the expression of AQPs in the intestines of mice. The expression of the genes for AQP 1 (A), 4 (C), 8 (E) and 11 (F) in the intestines was decreased by 5-FU. No significant difference in the gene expression of AQP 3 (B) and 7 (D) was observed in the different parts of the colon. Each column represents the mean ± S.E.M. of 3–6 independent experiments. *p<0.05, **p<0.01 and ***p<0.001 vs. vehicle (saline). G) The levels of AQP 4 and 8 protein in the distal colon from vehicle- and 5-FU-treated mice. Typical photographs of bands for AQP4 and 8 and GAPDH (left panel). The expression levels of AQPs were calculated as the ratios of the intensities of AQP to GAPDH proteins and are summarized in the right panel. Values are the means ± S.E.M. of 4 independent experiments. *p<0.05 and **p<0.01 vs. vehicle (saline).

In mice that had been treated with 5-FU, the gene expressions of AQPs 1 and 11 were significantly decreased in all of the intestines (p<0.05, Figure 6A/p<0.001, Fig. 6F), and the expressions of AQPs 4 and 8 were also significantly attenuated in the colons (p<0.001, Figures 6C and E). The gene expressions of AQPs 3 and 7 in the jejunum and ileum were not affected by 5-FU (Figures 6B and D). To confirm the 5-FU-induced downregulation of colonic protein levels of AQPs, immunoblotting was performed. AQPs 4 and 8 were significantly decreased by the administration of 5-FU (p<0.05 and p<0.001, Figure 6G).

**Figure 7:**
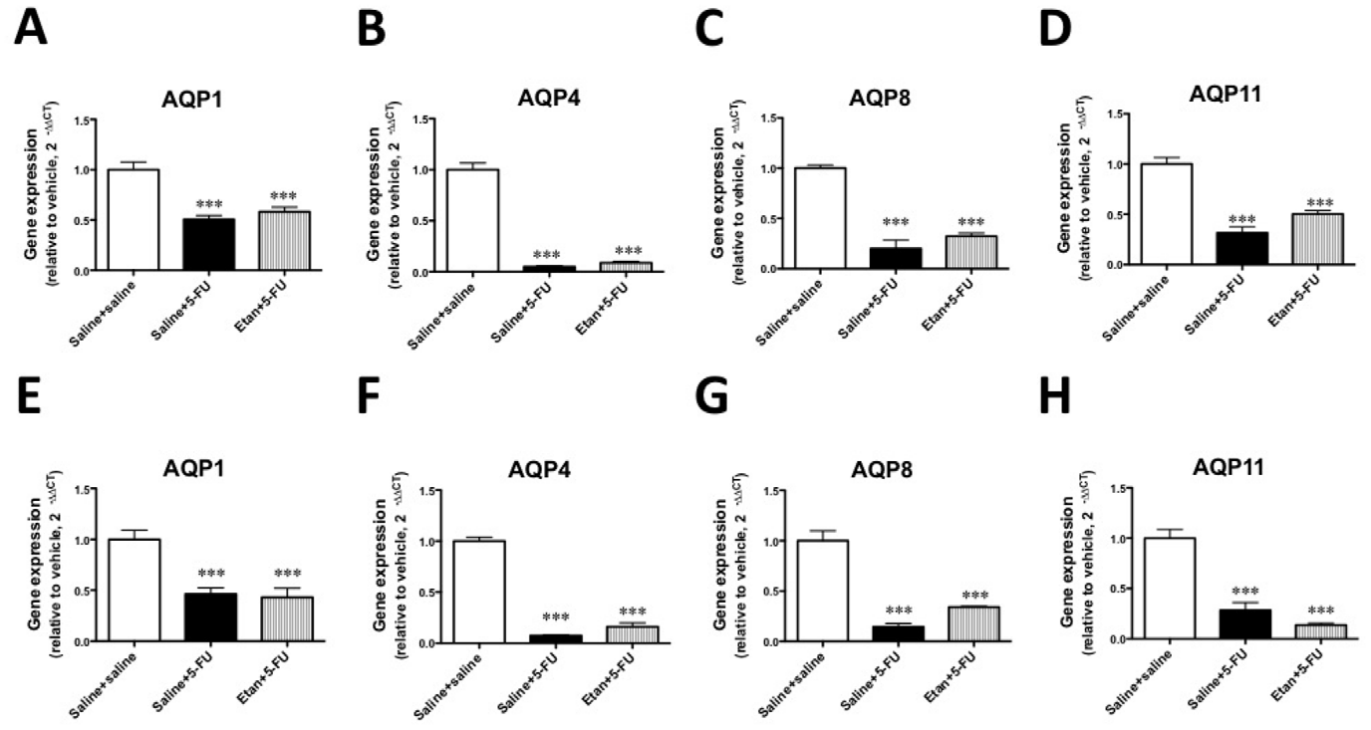
Effects of etanercept on the 5-FU-induced downregulation of aquaporin gene expression in the colons of mice. The 5-FU-induced downregulation of AQPs 1 (A and E), 4 (B and F), 8 (C and G) and 11 (D and H) in the transverse (A–D) and distal (E–H) colons was not recovered by the administration of etanercept. Each column represents the mean ± S.E.M. of 4–6 independent experiments. **p<0.01 and ***p<0.001 vs. Saline+saline. ^#^p<0.05, ^##^p<0.01 and ^###^p<0.001 vs. Saline +5-FU.

To investigate the interaction between the TNF-α pathway and the gene expression of AQPs, we examined the effect of the administration of etanercept on the 5-FU-induced decreases in the expressions of AQPs in the transverse and distal colons. The gene expressions of AQPs 1, 4, 8 and 11 were decreased in the transverse and distal colons of mice treated with 5-FU. However, these decreases in the gene expression of AQPs were not reversed by etanercept (Figure 7).

## Discussion

To investigate whether the development of 5-FU-induced diarrhea is associated with an altered gene expression of inflammatory cytokines in the intestines, we examined the changes in the gene expressions of TNF-α, IL-1β, IL-6, IFNγ, IL-17A and IL-22 in the small and large intestines of C57BL/6J mice with 5-FU-induced diarrhea. In the present study, we found that the expression of mRNAs encoding TNF-α, IL-1β, IL-6 IL-17A and IL-22 was dramatically increased in the proximal, transverse and distal colons of mice that exhibited severe diarrhea following the administration of 5-FU. In contrast, the gene expression of IFNγ, which has been classified as a T helper 1 (Th1)-type cytokine, was significantly increased only in the colon of mice treated with 5-FU.

Several treatments, such as the use of TNF-α inhibitors, seek to suppress these immune regulators in IBD, which is characterized as the chronic relapse of inflammatory disorders of the GI tract associated with an increased expression of IFNγ, IL-17A and IL-22 in inflamed mucosa [30], [31]. Therefore, we examined the effect of etanercept, which is a dimeric fusion protein consisting of the extracellular ligand-binding portion of the human 75 kDa (p75) TNF-α R linked to the Fc portion of human immunoglobulin G1 (IgG1), and which can bind to two TNF-α molecules to block their interaction with cell-surface TNF-α Rs and render TNF-α biologically inactive, on the 5-FU-induced upregulation of inflammatory cytokines. Interestingly, etanercept significantly reduced the 5-FU-induced increase in gene expression levels of IL-1β, IL-6, IFNγ, IL-17A and IL-22 in the colon, which suggests that a TNF-α pathway modulates the subsequent expression of these inflammatory cytokines. These findings are consistent with previous studies in uveoretinitis, rheumatoid arthritis and psoriasis vulgaris [32], [33], [34]. However, the 5-FU-induced loss of body weight was not recovered by the administration of etanercept. Furthermore, 5-FU-induced diarrhea was slightly, but not significantly, exacerbated by etanercept, indicating that an increase in inflammatory cytokines in different parts of the colon may not be directly associated with 5-FU-induced diarrhea. Therefore, we next examined the gene expression of AQPs in the intestines of mice that were treated with 5-FU.

In the present study, we found that the genes for AQPs 1, 3, 4, 7, 8 and 11 were expressed in the intestines of mice. At least seven AQP subtypes (AQPs 1, 3, 4, 5, 7, 8, 9 and 11) have been reported to be expressed in the GI tract and play an important role in several physiological and pathological processes [7], [10], [14]. In particular, the colon is a major site for the expression of AQPs 1, 3, 4 and 8 [10], [14] and there is some evidence regarding the physiological roles and functions of AQPs 1, 3, 4 and 8 in the colon. The water content of defecated stool is higher in AQP 4 knockout mice [35], and the inhibition of AQP 8 expression by small interfering RNA decreases osmotic water permeability [7]. On the other hand, AQP1 null mice exhibit only defective processing of dietary fat. In addition, both AQP1 null mice and AQP3 null mice do not appear to have a deficiency in colonic fluid transport [17], [36]. There is little information available on the function of other AQP subtypes in the intestine. There have been some reports on the physiological roles of AQPs in water absorption by the colon. Yang et al. [37] observed only minor phenotypic differences between wild-type and AQP8 null mice with regard to intestinal fluid transport, suggesting that compensatory changes in the expression of other water or solute transporters in AQP 8 null mice might account for their unremarkable phenotype. In contrast, AQP 4 null mice have been found to excrete feces with an elevated water content [35]. The transepithelial water permeability of the proximal colon of wild-type mice is higher than that of the distal colon of wild-type mice, and is also higher than that of the proximal colon of AQP 4 null mice. AQP 4 deletion does not affect water permeability in the distal colon, which results in an increase in the water content of defecated stool. These findings imply that AQP4 plays a role in transcellular movement of water across surface colonocytes [35]. Furthermore, the inhibition of AQP 8 expression by small interfering RNA significantly decreased water absorption in the rat proximal colon [38]. Thus, AQP 4 and AQP8 may play important roles in water absorption in the colon, particularly in the proximal colon, and decreased AQP expression might lead to an insufficiency of water absorption in this region of the GI tract. Changes in intestinal tissue levels of various AQPs have been reported in animal models and human diseases [26]. The expression of AQP 4 and AQP 8 mRNA is significantly decreased immediately after exposure to dextran sodium sulphate (DSS) in a C57BL/6 mouse model of colitis. Protein expression followed a pattern similar to that observed for AQPs mRNA. The rapid decrease in AQP 4 and AQP 8 expression is associated with changes in colonic water transport, leading to a shift in fluid flux from an absorptive state to a secretory state. Furthermore, colonic fluid transport returns to an absorptive state with an increase in AQP 4 and AQP 8 expression following the cessation of DSS, which suggests that there may be a functional correlation between fluid transport and AQP expression in the colon. These results from a model of DSS colitis in the mouse correlate with data from clinical investigations of human IBD. On the other hand, patients with active ulcerative colitis, Crohn’s colitis or infectious colitis who develop severe diarrhea show similar reductions in AQP 8 expression. To investigate whether the development of 5-FU-induced diarrhea is associated with an altered gene expression of AQPs in the intestines, we examined the changes in the gene expression of AQPs in the small and large intestines of mice with 5-FU-induced diarrhea. In the present study, the expression of mRNAs encoding AQPs 1, 4, 8 and 11 was decreased in the proximal, transverse and distal colons of mice that exhibited diarrhea following the administration of 5-FU. To further investigate the changes in AQP expression associated with inflammation along with changes in cytokines, and the possible correlation between altered AQP expression and diarrhea, we examined the effect of etanercept on the 5-FU-induced decrease in AQP gene expression in the intestine. As a result, the 5-FU-induced downregulation of AQP 1, 4, 8 and 11 was not recovered by the administration of etanercept, suggesting that altered AQP expression might be independent of TNF-α pathway-related inflammation in the intestines following the administration of 5-FU. These findings support the idea that the reduction of AQP by the administration of 5-FU may result in the dysfunction of water absorption in different regions of the colon, which in turn causes diarrhea. Especially, our findings regarding the altered expression of AQP 4 and AQP 8, both of which are considered to play important roles in water absorption in the colon, could, at least in part, explain the induction of diarrhea following the administration of 5-FU. Smith et al. [39] demonstrated that intraperitoneal administration of 5-FU increased myeloperoxidase in the intestine and suggested that the neutrophil could play an important role in the 5-FU-induced intestional inflammation. Taken together, we speculate that neutorophilic inflammation, but not Th1/Th17-type inflammations, may cause the impaired AQPs expression. Although further studies need to resolve this point, application of neutrophil elastase inhibitor such as sivelestat sodium may have effect on the impaired AQPs in the colon of the 5-FU treated mouse, (Page 16, lines 3–10).

In conclusion, in vivo administration of 5-FU caused increases in the gene expression of inflammatory cytokines (such as, TNF-α, IL-1β, IL-6, IFNγ, IL-17A and IL-22) and decreases in the expression of AQPs 1, 4, 8 and 11 in the colons of mice. Furthermore, the increased expression of cytokines was significantly reduced by s.c. administration of the TNF-α inhibitor etanercept, which did not counteract 5-FU-induced diarrhea, whereas etanercept did not restore the 5-FU-induced reduction in AQPs 1, 4, 8 and 11. The present study provides evidences that 5-FU administration develops both TNF-α modulating inflammation and decreased AQP expression in the intestine. However these pathophysiological conditions may be independent events. More importantly, intestional inflammation may not be directly associated with the pathological state of the 5-FU-induced diarrhea. Although further experiments are still needed, our findings raise the concept of a “TNF-α modulating cytokine-independent mechanism” for the diarrhea caused by 5-FU. We finally hypothesize here that impaired AQP expressions may predispose to intestional abnormality, such as severe diarrhea in response to 5-FU.

## Supporting Information

Table S1 **PCR primers used in the present study.**
(TIFF)
